# Chromosome-level genome assembly of the Japanese sparrowhawk *Accipiter gularis* (Aves: Accipitriformes: Accipitridae)

**DOI:** 10.1038/s41597-026-07743-w

**Published:** 2026-06-28

**Authors:** Wan Chen, Jiajie Li, Site Luo, Pengcheng Wang, Peng Li, Qing Chang, Chaochao Hu

**Affiliations:** 1 College of Environment and Ecology, Jiangsu Open University, Nanjing, 210002 China; 2 School of Life Sciences, Nanjing Normal University, Nanjing, 210023 China; 3 School of Life Sciences, Xiamen University, Xiamen, 361102 China; 4 Herpetological Research Center, College of Life Sciences, Nanjing Normal University, Nanjing, Jiangsu 210023 China

## Abstract

We present a high-quality chromosome-level genome assembly of the Japanese sparrowhawk (*Accipiter gularis*), a migratory raptor widely distributed across East Asia. The genome was assembled using a combination of Single-Tube Long Fragment Read (stLFR) sequencing and Hi-C chromatin conformation capture, resulting in a 1.19 Gb genome with a scaffold N50 of 43.17 Mb. Genome completeness assessment using Benchmarking Universal Single-Copy Orthologs (BUSCO) recovered 97.2% of conserved avian genes, indicating a high level of completeness. A total of 20,808 protein-coding genes were predicted, of which 92% were functionally annotated based on multiple public databases. Repetitive elements accounted for 7.95% of the genome, with retroelements forming the major component. Chromosome-level scaffolding enabled the anchoring of 98.9% of the assembled sequences onto 33 chromosomes, including 31 autosomes and the Z and W sex chromosomes. Comparative synteny analysis with *A. gentilis* revealed a high degree of chromosomal conservation, supporting the structural accuracy of the assembly. All raw sequencing data, genome assembly, and annotation files have been deposited in public repositories. This chromosome-level genome assembly provides a valuable genomic resource for studies of raptor evolution, migration ecology, population genomics, and comparative analyses within Accipitridae.

## Background & Summary

The Japanese Sparrowhawk (*Accipiter gularis* Temminck & Schlegel, 1844) is a small diurnal raptor belonging to the family Accipitridae. It is widely distributed across East Asia and represents a relevant system for studying avian migration, phenotypic variation, and population differentiation^1^. Based on morphological and molecular evidence, three subspecies are currently recognized: the widespread migratory *A. g. gularis*, the resident of conservation-concern *A. g. iwasakii* endemic to the Ryukyu and Yaeyama Islands, and *A. g. sibiricus*, distributed from Mongolia through northeastern China and exhibiting genetic differentiation in recent phylogeographic studies^2^. This species undertakes long-distance seasonal migration along the East Asian-Australasian Flyway, a major global migratory system linking breeding areas in East Asia with non-breeding regions in Southeast Asia and Australasia^3^. Two primary migration routes have been described: (1) a coastal corridor along mainland China, accounting for the majority of observed migratory individuals; (2) an island-based route via Japan and the Philippines^4^. Field observations consistently indicate that the coastal route supports the dominant migratory flux, with peak passage occurring during spring and autumn migration periods^1^.

In addition to migratory behavior, *A. gularis* exhibits notable intraspecific variation in molt strategies. Migratory populations generally follow a sequential molt pattern, whereas the resident subspecies *A. g. iwasakii* may exhibit suspended molt under unfavorable environmental conditions, as suggested by specimen-based and field observations^1^. The mechanisms and evolutionary drivers underlying these life-history differences remain poorly understood.

High-quality reference genomes provide essential resources for investigating evolutionary history, demographic processes, and the genetic basis of adaptive traits in non-model avian species^5^. However, genomic resources for Accipitriformes, particularly within Accipitridae, are still scarce. There are 405 genome assemblies from 248 raptor-related species in NCBI Datasets, but only 20 assemblies from 19 species are chromosome-level with gene annotation, and no annotated chromosome-level nuclear genome is currently available for *A. gularis* (retrieved on 2026.01.29)^6^. This limitation constrains comparative genomic analyses and reduces the resolution of evolutionary inference in this group.

Here, we present a chromosome-level genome assembly for *A. gularis*. The assembly was generated by integrating Single-Tube Long Fragment Read (stLFR) sequencing with Hi-C chromatin conformation data for scaffolding. The final assembled genome spans ~1.19 Gb, comprising 31 autosomes together with the Z and W sex chromosomes, with a scaffold N50 of 43.19 Mb. Genome completeness assessed using the aves_odb12 BUSCO dataset reached 97.2% (6,066 single-copy and 12 duplicated genes), with 1.7% fragmented and 1.1% missing.

Gene annotation identified 20,808 protein-coding genes, of which 92% were functionally annotated based on sequence homology, conserved domains, and Gene Ontology terms. Repetitive element account for 7.86% of the genome, with long terminal repeat (LTR) elements representing the predominant class. This chromosome-level genome assembly provides a foundational resource for comparative genomics, population genetic analyses, and studies of adaptive evolution in raptors. It also enables future investigation of the genetic basis of key traits in *A. gularis*, including migration, molt strategies, and environmental adaptation.

## Methods

### Ethics statement

All animal handling and experimental procedures were conducted in accordance with institutional and national guidelines for the care and use of animals. The study was approved by the Animal Care and Use Committee of Nanjing Normal University (approval ID: IACUC–2024257).

### Sample collection

A female *A. gularis* was collected at Nanjing Lukou International Airport, Jiangsu Province, China (31°44′4.53″ N, 118°52′15.13″ E). The specimen was assigned voucher number NNU-ACE-0148 and deposited at Nanjing Normal University. Tissue samples were dissected, briefly rinsed with sterile phosphate-buffered saline (PBS), and blotted dry. Tissues designated for DNA and RNA extraction were immediately flash-frozen in liquid nitrogen and stored at −80 °C until further processing. High molecular weight genomic DNA was extracted from pectoral muscle tissue using the DNeasy Blood & Tissue Kit (Qiagen, Hilden, Germany) following the manufacturer’s protocol. DNA integrity was assessed by 1% agarose gel electrophoresis, and DNA concentration was measured using a Qubit 4.0 fluorometer (Thermo Fisher Scientific, USA).

### Genome sequencing

For whole-genome sequencing, a Single-Tube Long Fragment Read (stLFR) library was constructed using the MGIEasy stLFR Library Prep Kit (MGI Tech Co., Ltd., Shenzhen, China) following the manufacturer’s instructions^7^. The library was sequenced on the DNBSEQ-T7 platform, generating 219.66 Gb of stLFR data (~188× coverage; Table 1). For genome size estimation, an additional 150 bp paired-end short-insert library was prepared and sequenced on the same platform, yielding 64.54 Gb of short-read data (~55× coverage; Table 1).

**Table 1:** Summary statistics of sequencing data, genome assembly, and annotation for Accipiter gularis.

Assembly parameter	Result
Short-insert paired-end reads	64.54 Gb
stLFR sequencing data	219.66 Gb
Hi-C sequencing data	119.86 Gb
RNA-seq data	7.20 Gb
Assembled genome size	1.196 Gb
Total number of contigs	31103
Contig N50	113.11 Kb
Total number of scaffolds	7402
Scaffold N50	43.17 Mb
Longest scaffold	84.19 Mb
Total length of anchored chromosomes	1.183 Gb
Anchored chromosomes rate	98.90%
Number of chromosomes	33 (31 autosomes + Z/W)
Predicted gene models	20,808
Repetitive elements	94.021 Mb (7.86%)
BUSCO (aves_odb12)	C: 97.2% [S: 97.0%, D: 0.2%], F: 1.7%, M: 1.1%

Note: BUSCO (Benchmarking Universal Single-Copy Orthologs) completeness is reported as: C (complete BUSCOs), including S (single-copy) and D (duplicated), F (fragmented), and M (missing), based on the aves_odb12 dataset.

For chromosome-level scaffolding, a Hi-C library was constructed from pectoral muscle tissue. Briefly, tissues were cross-linked with 1% formaldehyde at room temperature for 10 min, and the reaction was quenched with glycine. Nuclei were isolated and the chromatin was digested with the restriction enzyme *DpnII*, followed by proximity ligation to connect spatially adjacent DNA fragments. The ligated DNA was purified and used for library preparation with the NEBNext® Ultra™ II DNA Library Prep Kit (New England Biolabs, USA) according to the manufacturer’s instructions. The Hi-C library was sequenced on the DNBSEQ-T7 platform, generating 119.86 Gb of data (Table 1).

For transcriptome sequencing, total RNA was extracted from pectoral muscle tissue using the Animal Total RNA Kit (Omega Bio-Tek, Norcross, GA, USA) following the manufacturer’s instructions. RNA quality was assessed based on purity, integrity, and concentration prior to library construction. A strand-specific cDNA library was constructed and sequenced on the DNBSEQ-T7 platform (MGI Tech Co., Ltd., Shenzhen, China), generating 7.2 Gb of RNA-seq data (Table 1). In total, 219.66 Gb of stLFR data, 64.54 Gb of short-read data, 119.86 Gb of Hi-C data, and 7.2 Gb of transcriptome data were generated for downstream analyses (Table 1).

### Genome size estimation

**Fig. 1:**
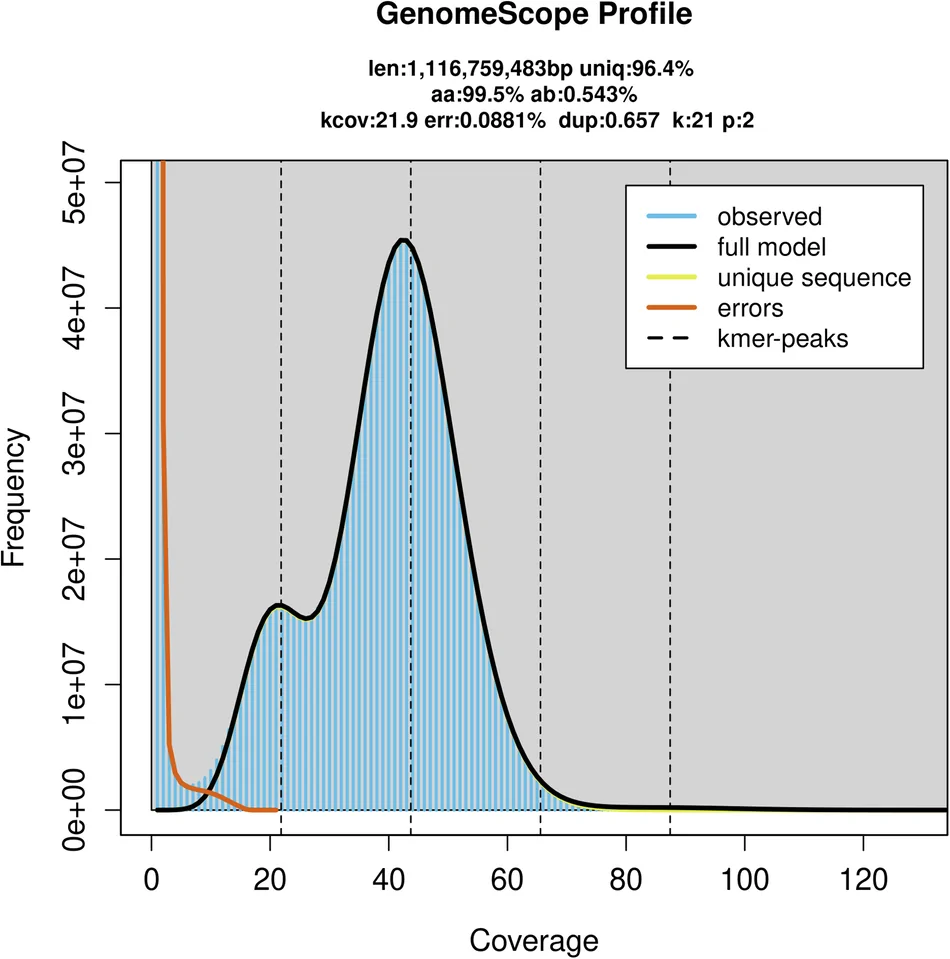
K-mer frequency distribution and GenomeScope model of the Accipiter gularis genome.

Raw short-read data were processed using fastp v 0.23.4^8^ to remove adapter sequences and low-quality reads. Genome size was estimated based on k-mer frequency analysis. Briefly, 21-mer counting was performed using Jellyfish v2.3.1^9^, and a k-mer frequency histogram was generated. The histogram was subsequently analyzed with GenomeScope 2.0^10^ to estimate genome size, heterozygosity, and repeat content. The genome size of *A. gularis* was estimated to be 1.116 Gb, with a heterozygosity rate of 0.547% (Fig. 1).

### Genome assembly

The reference genome was assembled by integrating stLFR sequencing data with Hi-C data for chromosome-level scaffolding. Prior to assembly, the stLFR dataset was converted into a LongRanger-compatible format using the stlfr2supernova pipeline^11^. *De novo* assembly was performed using Supernova pipeline v2.1.1 with default parameters^12^. The diploid assembly was exported in haploid representation using the *mkoutput* module. The initial assembly comprised 10,652 scaffolds, with a total length of 1.266 Gb and a scaffold N50 of 14.805 Mb. Potential contaminant sequences were identified and removed using FCS-GX v0.5.0 with default settings^13^.

**Fig. 2:**
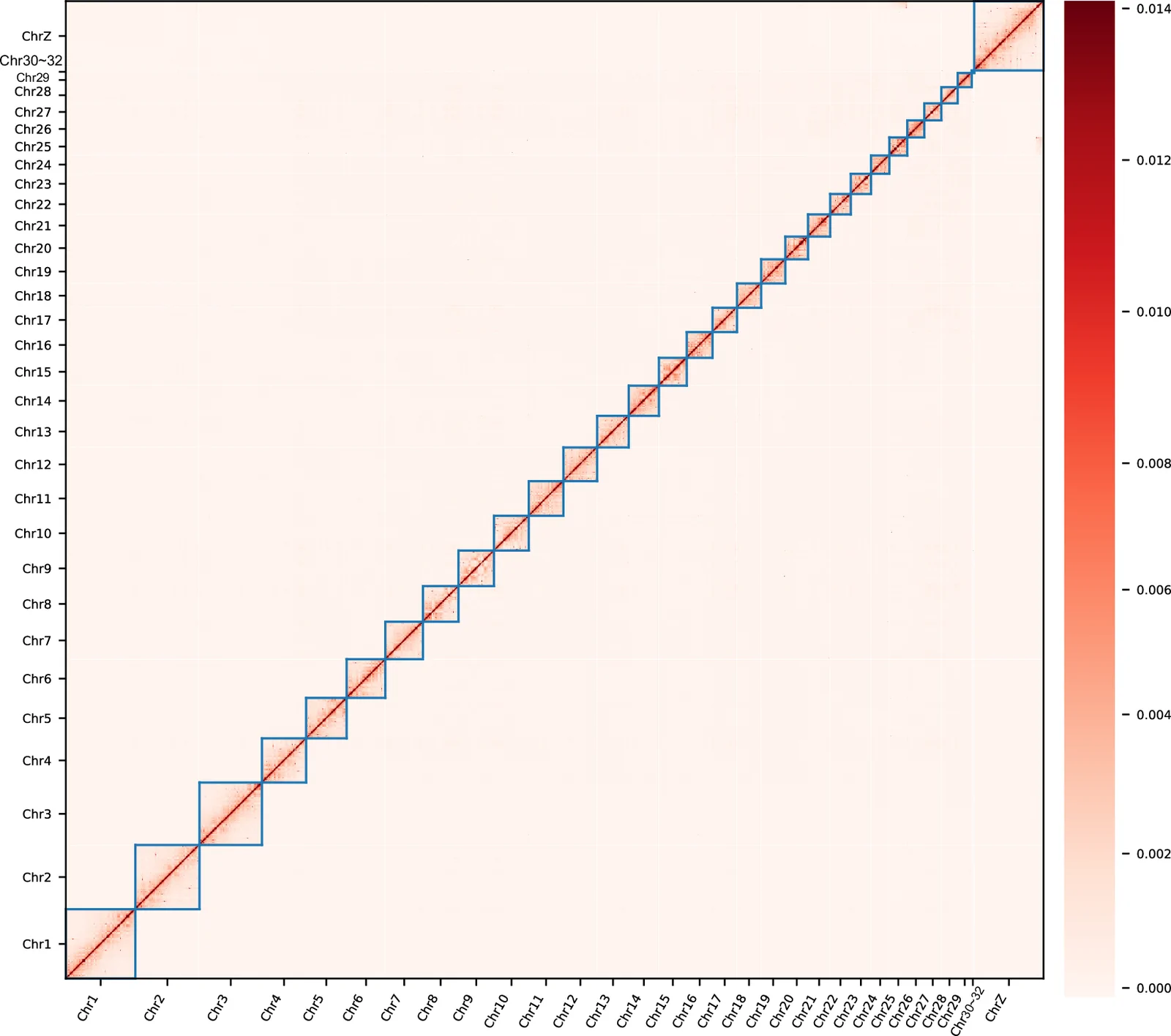
Hi-C contact map of the Accipiter gularis genome assembly. The heatmap shows chromatin interaction frequencies across assembled chromosomes, with interaction intensity indicated by a color gradient from white (low) to red (high). Strong interaction signals along the diagonal and clear boundaries between chromosomes support the accuracy of chromosome-level scaffolding. Chromosomes are ordered by size.

Hi-C data were used for chromosome-level scaffolding with ALLHiC v0.9.12^14^. Contig N50 was derived from the Supernova assembly output, and scaffold N50 was calculated after Hi-C scaffolding using ALLHiC, both based on the sorted length distribution of assembled sequences. The final assembly had a total length of 1.196 Gb and a scaffold N50 of 43.177 Mb. In total, 98.9% of the assembled bases were anchored to 33 chromosomes. The Hi-C contact maps showed clear chromosome-scale interaction patterns, supporting the accuracy of the scaffolding (Fig. 2).

**Fig. 3:**
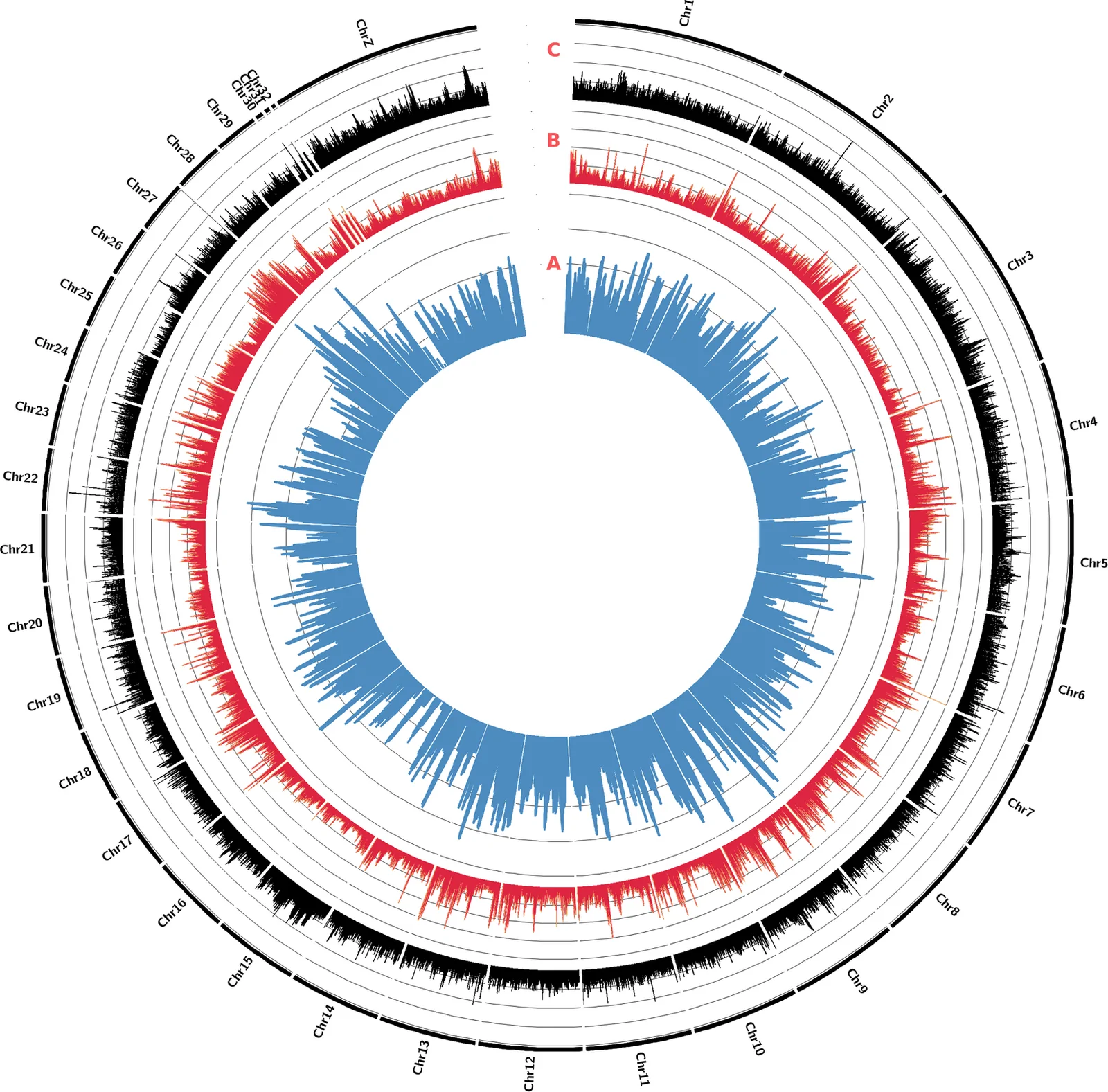
Circular representation of genomic features in the Accipiter gularis genome. From the innermost to the outermost ring, the tracks represent: (**A**) gene density, (**B**) GC content, and (**C**) repeat element density. All features were calculated in non-overlapping 200 Kb sliding windows.

Assembly completeness was evaluated using BUSCO v5.7.10^15^ with the *aves_odb12* dataset. In genome mode, the assembly recovered 97.2% of expected single-copy orthologs, including 97.0% complete and single-copy, 0.2% duplicated, 1.7% fragmented, and 1.1% missing genes, indicating a high level of completeness (Table 1; Fig. 3). In protein mode, BUSCO analysis based on the predicted protein set yielded comparable results, further supporting the high completeness and accuracy of gene annotation. Chromosomes were named and ordered according to their size following Hi-C scaffolding, and further validated by synteny analysis with the reference genome of *A. gentilis*, enabling assignment of chromosome homology and identification of sex chromosomes (Z and W).

### Repeat element annotation

Repetitive elements in the *A. gularis* genome were identified using a combined *de novo* and homology-based approach. A species-specific repeat library was first generated *de novo* using RepeatModeler v2.0.6^16^. The resulting consensus sequences were merged with known repeat sequences from Repbase (v20181026) to construct a custom repeat library. This library was then used by RepeatMasker v4.1.5 to identify and annotate repetitive elements across the genome^15^.

In total, 94.02 Mb of repetitive sequences were identified, accounting for 7.95% of the assembled genome (Table 2). Interspersed repeats represented the major fraction, spanning 83.76 Mb (7.08%). Retroelements accounted for 50.59 Mb (4.28%), whereas DNA transposons contributed 0.22 Mb (0.02%).

**Table 2:** Summary of repetitive elements identified in the Accipiter gularis genome.

Class	Copy number	Total length (bp)	% of assembly
**Retroelements (total)**	102,648	50,591,710	4.28
Penelope	852	136,775	0.01
LINEs	57,893	22,414,763	1.90
L2/CR1/Rex	57,041	22,277,988	1.88
LTR elements	44,755	28,176,947	2.38
Gypsy/DIRS1	217	58,562	0.00
Retroviral	25,752	18,230,358	1.54
**DNA transposons**	671	224,090	0.02
hobo-Activator	626	199,523	0.02
**Unclassified interspersed repeats**	120,696	32,941,029	2.79
**Total interspersed repeats**	–	83,756,829	7.08
**Simple repeats**	228,776	8,279,211	0.70
**Low-complexity regions**	44,473	1,985,365	0.17
**Total masked bases**	–	94,021,405	7.95

Note: LINEs, long interspersed nuclear elements; LTR, long terminal repeat; L2/CR1/Rex, a LINE family; Gypsy/DIRS1, a class of LTR retrotransposons; DNA transposons, class II transposable elements; hobo-Activator, a DNA transposon family.

Among interspersed repeats, long terminal repeat (LTR) elements were the most abundant, covering 28.18 Mb (2.38%), followed by long interspersed nuclear elements (LINEs), which occupied 22.41 Mb (1.90%). Within LINEs, the L2/CR1/Rex family was predominant (22.28 Mb, 1.88%). Retroviral-related elements accounted for 18.23 Mb (1.54%), while Penelope elements were rare.

In addition, 32.94 Mb (2.79%) of the genome was annotated as unclassified interspersed repeats, indicating that a substantial proportion of repetitive sequences could not be assigned to known repeat families. Simple repeats and low-complexity regions accounted for 8.28 Mb (0.70%) and 1.99 Mb (0.17%), respectively.

Overall, the *A. gularis* genome shows a relatively low proportion of repetitive sequences, dominated by retroelements and unclassified repeats, with LTR elements representing the most abundant classified repeat category.

### Gene prediction and functional annotation

Gene prediction was performed using an integrative approach combining *ab initio*, transcriptome-based, and homology-based approaches. For *ab initio* prediction, BRAKER v3.0.8 was used to integrate RNA-seq alignments and protein homology evidence, and gene models were refined using AUGUSTUS^17,18^. For transcriptome- based prediction, RNA-seq reads were aligned to the genome using HISAT2 v2.2.1^19^, and transcripts were assembled using StringTie v2.2.1^20^. Coding sequences (CDSs) were predicted using TransDecoder v5.5.0 (minimum ORF length ≥ 50 amino acids). CDSs supported by Pfam-A (via HMMER^21^, E ≤ 1 × 10^−5^) or Swiss-Prot (via DIAMOND^22^, E ≤ 1 × 10^−5^) were retained as high-confidence gene models.

For homology-based predictions, GeMoMa v1.9^23^ was used with protein datasets from *Gallus gallus* (GCF_016699485.2) and *A. gentilis* (GCF_929443795.1). All evidence sources were integrated using EVidenceModeler v2.0.0^24^, with weights assigned as follows: *ab initio* (AUGUSTUS) = 4, homology-based (GeMoMa) = 10, and transcriptome-based (StringTie + TransDecoder) = 12. This process yielded 20,808 high-confidence protein-coding gene models.

Functional annotation was performed by sequence similarity searches against the UniProt and NCBI non-redundant (NR) databases using DIAMOND blastp (E ≤ 1 × 10^−5^). Additional functional classification was conducted using eggNOG-mapper v2.1.12^25^, mapping genes to Gene Ontology (GO)^26^ and Kyoto Encyclopedia of Genes and Genomes (KEGG)^27^ databases. Protein domains and GO terms were further annotated using InterProScan v5.73 (parameters: -appl Pfam -iprlookup -goterms)^28^. Transfer RNAs were identified using tRNAscan-SE v2.0.1, and other non-coding RNAs were predicted using Infernal v1.1.5 in combination with the Rfam database^29^.

In total, 19,142 genes (92% of predicted protein-coding genes) were functionally annotated with at least one database-derived assignment (Table 3), providing a comprehensive resource for future functional, evolutionary, and comparative genomic analyses.

**Table 3:** Summary of functional annotation of predicted genes in the Accipiter gularis genome.

Database	Number of annotated genes	Proportion of gene models annotated (%)
GO	15,474	74%
KEGG	13,434	65%
KOG	16,441	79%
Pfam	15,426	74%
Swiss-Prot	17,583	85%
NR	19,069	92%
Total	19,142	92%

Note: GO, Gene Ontology; KEGG, Kyoto Encyclopedia of Genes and Genomes; KOG, Eukaryotic Orthologous Groups; Pfam, protein families database; Swiss-Prot, manually curated protein sequence database; NR, NCBI non-redundant protein database.

### Genome quality assessment

Assembly and annotation completeness were evaluated using Benchmarking Universal Single-Copy Orthologs (BUSCO v5.8.2)^15^ with the aves_odb12 dataset. Genome completeness assessed using BUSCO (aves_odb12 dataset) confirmed a high level of completeness, consistent with the results obtained during genome assembly (Table 1). Consistent with BUSCO results, the assembly exhibited high contiguity, with a scaffold N50 of 43.17 Mb and 98.9% of the genome anchored to 33 chromosomes.

**Fig. 4:**
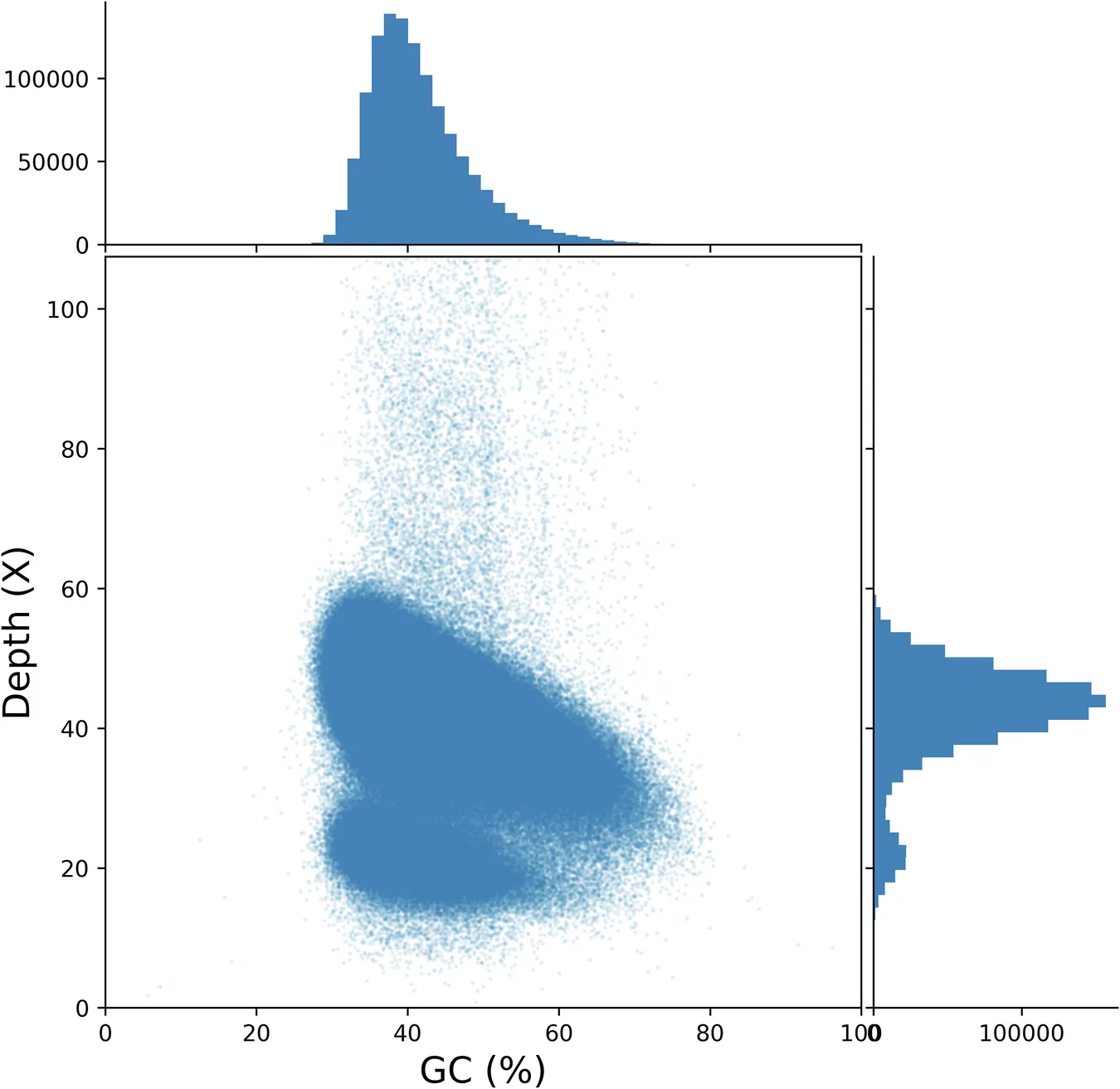
Distribution of GC content and sequencing depth across the Accipiter gularis genome. GC content and average sequencing depth were calculated in 1Kb windows, with each dot representing one genomic window. Mean GC content was 41.67% (95% range: 33.10–55.10%), and mean sequencing depth was 45.88X (95% range: 22.27–51.70X).

To assess assembly accuracy and potential contamination, clean short-insert paired-end reads were mapped back to the assembled genome using BWA v0.7.19^30^. The mapping rate reached 98.86%, with an overall genome coverage of 99.84%. GC content and sequencing depth across the genome were further examined to detect potential biases or contamination. The results showed uniform coverage across chromosomes and no evident GC bias relative to sequencing depth, indicating high assembly accuracy and the absence of detectable contamination (Fig. 4).

### Genome synteny analysis

**Fig. 5:**
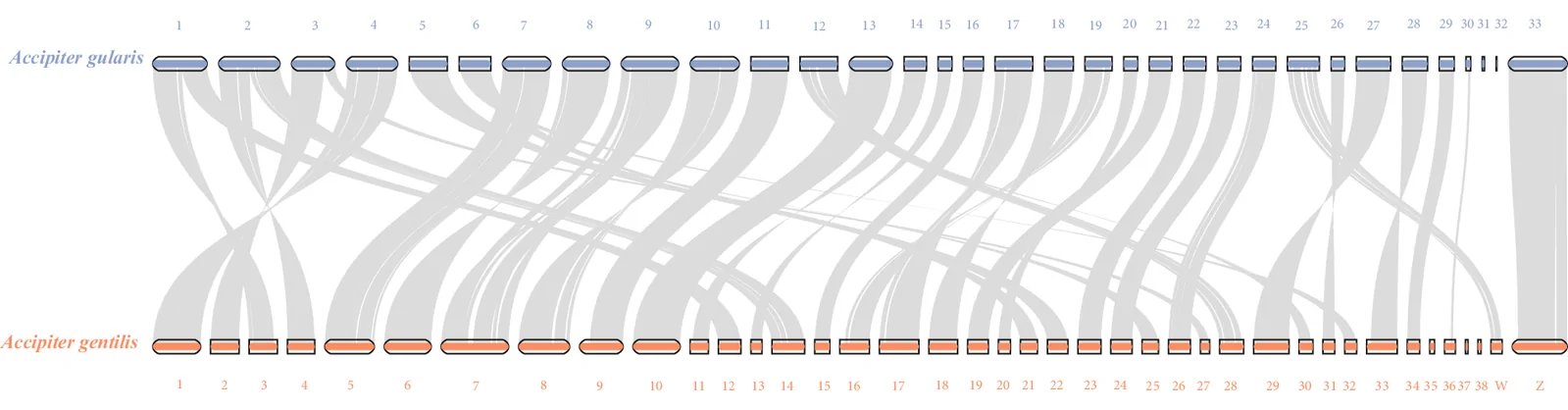
Chromosome-scale synteny between Accipiter gularis and A. gentilis. Chromosomes of A. gularis (top) and A. gentilis (bottom) are shown with corresponding homologous regions connected by grey links, indicating conserved genomic blocks. The high degree of collinearity between the two genomes supports the accuracy of chromosome-level assembly in A. gularis and enables the identification of homologous chromosomes, including the sex chromosomes (Z and W).

To evaluate chromosomal conservation and validate the structural accuracy of the assembly, whole-genome synteny analysis was performed between *A. gularis* and *A. gentilis*. Protein-coding genes from *A. gularis* were aligned to those of *A. gentilis* using BLASTP (BLAST+ v2.12.0) with an E-value cutoff of 1 × 10^−10^, retaining up to five target alignments per query^31^. The resulting alignments were processed with MCScanX to identify collinear gene pairs and syntenic blocks under default parameters^32^. Syntenic relationships were visualized using the JCVI package (formerly Python MCscan), which provides chromosome-scale comparative analysis^33^. The analysis revealed a high degree of conserved synteny between the two genomes, supporting the accuracy of chromosome-level scaffolding in *A. gularis*. Based on conserved collinearity with the annotated genome of *A. gentilis*, the Z and W sex chromosomes of *A. gularis* were confidently identified and assigned (Fig. 5).

## Data Records

All datasets generated in this study have been deposited in public repositories. Raw sequencing data, including stLFR, short-read, Hi-C, and RNA-seq datasets, are available in the NCBI Sequence Read Archive (SRA) under accession numbers SRR30682834–SRR30682836^34–36^, associated with BioProject PRJNA1161806^37^. The chromosome-level genome assembly of *A. gularis* has been deposited in NCBI GenBank GCA_046127185.1^38^. Genome annotation files, including gene models and functional annotation results, are available in the Figshare repository (https://doi.org/10.6084/m9.figshare.31866352)^39^.

## Technical Validation

Genome size was estimated using 21-mer analysis based on DNBSEQ short-read data, yielding an estimated genome size of 1.116 Gb and a heterozygosity rate of 0.547% (Fig. 1). This estimate is consistent with the final assembly size of 1.196 Gb (Table 1), indicating overall completeness of the genome assembly. The genome assembly showed high continuity, with a contig N50 of 113.11 Kb and a scaffold N50 of 43.17 Mb (Table 1). A total of 98.9% of the assembled sequences were anchored to 33 chromosomes. Hi-C contact maps displayed clear chromosome-scale interaction patterns, supporting the accuracy of the scaffolding (Fig. 2).

To evaluate assembly accuracy and potential contamination, Illumina short reads were mapped to the assembled genome using BWA v0.7.6. The mapping rate reached 98.86%, with an overall genome coverage of 99.84%. GC content distribution showed no obvious bias relative to sequencing depth, suggesting no detectable contamination or major sequencing artifacts (Fig. 4). Repeat annotation identified 94.02 Mb of repetitive sequences, accounting for 7.95% of the genome. Interspersed repeats accounted for 83.76 Mb (7.08%), dominated by retroelements and unclassified repeats, with LTR elements representing the most abundant classified repeat type (Table 2). Genome completeness assessed using BUSCO (aves_odb12 dataset) recovered 97.2% complete genes, including 97.0% single-copy and 0.2% duplicated BUSCOs, with 1.7% fragmented and 1.1% missing genes (Table 1). Overall, these results support a high-quality genome assembly with strong continuity, high completeness, and accurate chromosome-level scaffolding.

## Data Availability

All raw sequencing data generated in this study are available in the NCBI Sequence Read Archive (SRA) under BioProject accession PRJNA1161806^37^. The SRA runs are available under accession numbers SRR30682834–SRR30682836^34–36^. The chromosome-level genome assembly of *Accipiter gularis* has been deposited in NCBI GenBank under accession GCA_046127185.1^38^. Genome assembly and annotation files are also available in the Figshare repository (https://doi.org/10.6084/m9.figshare.31866352)^39^.

## Code availability

All bioinformatic analyses were performed using publicly available software, with default settings unless otherwise stated. No custom scripts were employed. The software and key parameters used in this study are described below.

**Genome size estimation**

1. Jellyfish v2.3.1: parameters: -m 21 -s 100M -U 500 -t 40.

2. GenomeScope 2.0: all parameters were set as default

**Genome assembly**

1. stlfr2supernova pipeline: fake_10x.pl script

2. Supernova v2.1.1: parameters: --id=SQY --fastqs=/media/datarun/SQY10x --maxreads=all, and the haploid representation was exported using supernova mkoutput --style=pseudohap --asmdir=/media/datarun/outs/assembly --outprefix=SQY

3. ALLHiC v0.9.12: parameters: `PreprocessSAMs.pl sample.bwa_aln.sam genome.fasta GATC`, `filterBAM_forHiC.pl sample.bwa_aln.REduced.paired_only.bam sample.clean.sam`, `ALLHiC_partition -b sample.clean.bam -r genome.fasta -e GATC`, `allhic extract sample.clean.bam genome.fasta --RE GATC`, `allhic optimize sample.clean.counts_GATC.txt sample.clean.clm`, and `ALLHiC_build genome.fasta`

4. BUSCO v5.7.10: parameters: -l busco_downloads/lineages/aves_odb12

**Repeat annotation**

1. RepeatModeler v2.0.6: parameters: - RepeatModeler -pa 96 -database genome -engine ncbi -LTRStruct

2. RepeatMasker v4.1.5: parameters: -pa 40 -lib hap2.after_sorter.fa -no_is -xsmall -poly -html -gff -dir masker2 hap2_rename.fasta

**Gene prediction**

1. TransDecoder v5.5.0: coding sequence prediction (minimum ORF ≥50 aa)

2. GeMoMa v1.9: parameters: threads=96, GeMoMa.Score=ReAlign, and AnnotationFinalizer.r=NO

3. EVidenceModeler v2.0.0: parameters: AUGUSTUS = 4, GeMoMa = 10, and StringTie + TransDecoder = 12.

**Functional annotation**

1. DIAMOND blastp: sequence similarity search against the UniProt and NR databases (E ≤ 1 × 10^−5^)

2. eggNOG-mapper v2.1.1: parameters: emapper.py -d euk -i pep.fasta -o eggNOG_hmmer --cpu 95 --usemem --no_file_comments

3. InterProScan v5.73: parameters: interproscan.sh -i pep.fasta -f tsv,xml --goterms --iprlookup --pa -cpu 96

**Genome quality assessment**

1. BWA v0.7.6: parameters: bwa mem -t 96 -R '@RG\tID:Survey\tSM:Survey\tPL:ILLUMINA' ${REF} ${R1} ${R2} | samtools view -@ 8 -bS - | samtools sort -@ 8 -o ${PREFIX}.sorted.bam.
